# Fall risk prediction using temporal gait features and machine learning approaches

**DOI:** 10.3389/frai.2024.1425713

**Published:** 2024-08-28

**Authors:** Zhe Khae Lim, Tee Connie, Michael Kah Ong Goh, Nor ‘Izzati Binti Saedon

**Affiliations:** ^1^Faculty of Information Science and Technology, Multimedia University, Melaka, Malaysia; ^2^Department of Medicine, Faculty of Medicine, Universiti Malaya, Kuala Lumpur, Malaysia

**Keywords:** fall risk prediction, human pose estimation, machine learning, computer vision, gait features

## Abstract

**Introduction:**

Falls have been acknowledged as a major public health issue around the world. Early detection of fall risk is pivotal for preventive measures. Traditional clinical assessments, although reliable, are resource-intensive and may not always be feasible.

**Methods:**

This study explores the efficacy of artificial intelligence (AI) in predicting fall risk, leveraging gait analysis through computer vision and machine learning techniques. Data was collected using the Timed Up and Go (TUG) test and JHFRAT assessment from MMU collaborators and augmented with a public dataset from Mendeley involving older adults. The study introduces a robust approach for extracting and analyzing gait features, such as stride time, step time, cadence, and stance time, to distinguish between fallers and non-fallers.

**Results:**

Two experimental setups were investigated: one considering separate gait features for each foot and another analyzing averaged features for both feet. Ultimately, the proposed solutions produce promising outcomes, greatly enhancing the model’s ability to achieve high levels of accuracy. In particular, the LightGBM demonstrates a superior accuracy of 96% in the prediction task.

**Discussion:**

The findings demonstrate that simple machine learning models can successfully identify individuals at higher fall risk based on gait characteristics, with promising results that could potentially streamline fall risk assessment processes. However, several limitations were discovered throughout the experiment, including an insufficient dataset and data variation, limiting the model’s generalizability. These issues are raised for future work consideration. Overall, this research contributes to the growing body of knowledge on fall risk prediction and underscores the potential of AI in enhancing public health strategies through the early identification of at-risk individuals.

## 1 Introduction

In recent years, falls have been recognized as a significant public health issue in society. The issue is rapidly growing, especially among older adults. According to the report from the World Health Organization (WHO) in 2021, an estimated 684,000 individuals globally die from falls each year, with over 80% of these deaths happening in poor and middle-income countries. Additionally, an estimated 37.3 million falls require medical attention. Moreover, individuals aged 60 and above exhibit the highest fatality rate from falls (World Health Organization, 2021). Therefore, early detection of individual fall risk is crucial for mitigating public concerns.

The current methods for reliably assessing an individual’s risk of falling are clinical fall risk assessment tools. However, these methods rely on physical tests, surveys, and many other procedures, which could be both costly and time-consuming. They may strain hospital staff resources.

Recently, researchers have turned to artificial intelligence to identify human fall risks. They have discovered a connection between falls and gait, leading to the utilization of wearable sensors for gait assessment and machine learning techniques in the field. However, wearable sensors have constraints such as inconvenience for participants as well as high costs for the devices.

This study aims to discover suitable gait features acquired from camera sources that can be used to predict falls effectively. The methodology involved data collection through the Timed Up and Go (TUG) assessment, referred to as the MMU-FRiP dataset (Time Up and Go (TUG), 2024b). Computer vision and machine learning methods are applied to estimate the human pose and extract gait features extraction that can be indicative of falls. During the process, individuals are divided into two categories: Faller and Non-faller. Faller refers to people who are at low risk, whereas non-fallers are at high risk. Surprisingly, gait features extracted using simple machine learning approaches performed well in the study by achieving promising performance, indicating their suitability for identifying individuals at risk of falling. Specifically, we found a high association between falls and increased cadence, and shorter step times. The results showed that gait analysis using artificial intelligence techniques shows promise in automated fall risk assessment.

## 2 Related work

Falling among the elderly has gained international recognition as a serious public health issue. Many academics have started to investigate fall risk assessment to create workable solutions that would lessen the issues. Older adults’ fall risk is generally correlated with their mobility, and this section addresses the relevant studies on fall risk prediction in this age range.

### 2.1 Conventional methods

In 2022, a group of researchers (Chakraborty and Sorwar, 2022) proposed a method that could automatically identify fall risk in elderly individuals using statistical and machine learning approaches. The study assessed a long-term mobility monitoring dataset from 71 older people and divided them into a group of fallers or non-fallers. The methods involved three different approaches that were tested and two different machine learning algorithms for classification, AdaBoost and Decision Tree, as well as cross-validation (20 or 30-fold) to achieve the mean AUC score, and the mean AUC score was used as a performance comparison. The first approach does not involve any feature selection or data shuffling, then the second approach uses data shuffling without any feature selection, as well as the third approach uses both data shuffling and feature selection. The study’s findings demonstrate that the third approach with a decision tree classifier achieved the highest mean AUC score of 0.98, highlighting that the random data shuffling combined with cross-validation can significantly improve classification accuracy. However, the study reveals that certain features did not perform well in fall risk classification and the dataset for the research is limited.

Another group of researchers (Beauchet et al., 2018) conducted a study on fall predictions using artificial neural network (ANN) approaches. The data were gathered from 848 older inpatients with different kinds of characteristics, such as age, gender, number of drugs taken daily, use of psychoactive drugs or analgesics, history of previous falls, ability to stand, ability to sit and to stand, ability to stand up and sit down, mobility disorders, neuropsychiatric disorders, and so on. In the study, participants were categorized into two groups, non-fallers, and fallers. Three ANN algorithms were applied in the process, including MLP, averaged neural network, and NEAT. The dataset was split into a ratio of 80:20 for training and testing sets. In the training phase, all classifiers achieve impressive results, while MLP has the greatest accuracy of 99.71%. While in the testing phase, the averaged neural network had a poor result of 17.86%, while MLP and NEAT achieved promising results of 81.44% and 83.83%, respectively. However, the study mentioned there is a limitation on dataset availability, which might have an impact on the algorithm’s ability to generalize effectively.

Later on, a study (Velusamy et al., 2023) provided detailed information on real-time fall prediction using wearable sensors and machine learning algorithms. The study discussed multiple machine learning algorithms and sensor modalities that had been used for the fall risk assessment, such as decision trees, support vector machines (SVM), neural networks, random forests, and logistic regression algorithms, as well as wearable sensors such as accelerometers, gyroscopes, magnetometers, and pressure sensors. Several datasets were collected through the fall risk assessment using wearable sensors, including FARSEEING, UP-Fall, WISDM, UVA-Fall, and MobiAct. Those datasets contained the details of gait characteristics, daily activities, and falls. Overall, the results of the study indicated the effectiveness of machine learning approaches for fall prediction assessments according to the accuracy score they achieved. The study also identified the difficulties and constraints of wearable sensors for fall risk, including sensor positioning and dependability.

In 2022, an analysis was conducted to predict the risk of falls in the elderly using a single inertial measurement unit on the lower back by estimating spatio-temporal gait parameters (Aqueveque et al., 2022). The process included feature selection, characterization, and machine training. The analysis focused on the gait cycle parameters of fallers and non-fallers. Then, the dataset consisted of mobility data from 69 subjects, 31 of them were fallers and 38 were non-fallers. The gait parameters contained the data of participants’ initial foot contact (IC), last foot contact (FC), cadence, step time, and stride time. The classification algorithms used were SVM with a polynomial kernel, Random Forest, and the F1-Score for performance comparison. The results showed that the SVM classifier with a third-degree polynomial kernel had an accuracy of 59%, recall of 91%, and F1-score of 71% in identifying subjects at risk of falling. On the other hand, the RF classifier also showed promising results (accuracy = 81–98%, F1-score = 70%) but did not exceed the performance of the SVM classifier (F1-score). The study’s shortcomings included a small sample size that prevented generalization to the entire elderly population and the use of an external database.

Besides, research (Ta and Jin, 2023) performed fall risk prediction using multiple machine learning approaches such as logistic regression, KNN, RF, SVM, and K-means cluster. The study used 354 datasets from tests on the Berg Balance Scale, which is one of the clinical fall risk assessment tools. The Berg Balance Scale consists of three risk categories: low risk (score range: 41–56), medium risk (score range: 21–40), and high risk (score range: 0–20). The tests included sitting to standing, standing unsupported, sitting unsupported, standing to sit, transfers, standing eyes closed, standing feet together, reaching forward, retrieving objects, turning to look, turning 360, foot on stool, standing tandem, and stand on one leg. In the end, the results of the ML models able to achieve impressive accuracy ranged between 92.33% and 97.89% for each subset tested. However, there is a limitation mentioned in the study, which is the limited patient dataset, which leads to bias.

Apart from that, a study was performed on fall risk prediction in Parkinson’s disease using real-world inertial sensor gait data (Ullrich et al., 2023), to compare different data aggregation approaches and machine learning models for the prospective prediction of fall risk using gait parameters derived either from continuous real-world recordings or from unsupervised gait tests. The dataset contained real-world gait and unsupervised 4×10-Meter-Walking-Tests from 40 PD patients. Different classifier methods were used, including SVM, RF, and GB. The Random Forest classifier reported the highest balanced accuracy of 74.0% (sensitivity: 60.0%, specificity: 88.0%) when aggregating all walking bouts and days of each participant. However, due to the study’s unsupervised nature, several limitations arise. These include differences in the amount of data provided by participants, differences in the number of recordings and gait test executions per day, and missing recordings due to technical and usability difficulties encountered by participants when handling the recording.

Another study (Lockhart et al., 2021) was conducted involving fall risk detection among community-dwelling older adults using an IMU sensor. The study used 58 gait parameters as the dataset, including linear and nonlinear parameters, collected by 171 community-dwelling older adults during 10-meter walking tests at their normal speed. While 127 participants’ gait data was used for training the classification model, the rest will be used for testing. In this study, 3 different experiments were using an RF classifier. Experiment I covered the random forest base model development, validation, and blind testing, while Experiment II involved the development, validation, and blind testing of the random forest model with feature engineering. The use of linear gait variables vs. nonlinear gait variables was compared. Finally, Experiment III applied a random forest model with feature engineering and both linear and nonlinear variables. The results showed that Experiment III achieved the highest accuracy of 81.6 ± 0.7%. The study also pointed out a weakness that was related to the limited generalizability of the model due to the lack of patient-specific training data sets at the beginning of the data collection phase, which limited the model’s applicability to different populations.

In 2024, a study was conducted utilizing accelerometer data as well as a machine learning approach for fall risk prediction (Angsuwan et al., 2024). The dataset comprises accelerometer gait data and demographic information collected from 160 elderly people aged 60 to 86 years. The study also uses k-fold cross-validation to address the restricted dataset issue. In the data collection process, participants were given instructions to wear a 3-axis accelerometer sensor on their anterior waist and complete the TUG test twice. Overall, after deleting the noise sample, there were a total of 319 samples. The TUG evaluation then labeled each sample with its fall risk, classifying those with completion times of 13.5 s or more as fallers and the remaining ones as non-fallers. During the implementation phase, the accelerometer data is preprocessed using the moving average method. Next, model enhancement techniques like feature selection, PCA, and SMOTE oversampling are used. Overall, the RF model outperformed others in the experiment using the top 10 features and demographic data, scoring 0.98 on the AUC score.

Apart from that, a study (Nishiyama et al., 2024) proposed utilizing single gait cycle data and machine learning in elderly fall risk prediction. The dataset includes acceleration and rotational velocity data from an IMU collected from 44 participants (22 fallers) using a smartphone to record their process of walking for 6 s under a 10-meter walking path four times. The modeling method includes using inner and outer cross-validation to optimize hyperparameters. Finally, the gradient-boosting decision tree algorithm achieved an amazing mean accuracy of 0.936 in five-fold cross-validation, with age being the most important feature. The study also emphasizes the benefits of this new method, which only requires a gait cycle.

Another study (Kou et al., 2024) proposed the use of IMUs and machine learning approaches for fall risk assessment. The dataset contains 28 kinematic data collected from 28 workers aged 60 to 80 performing tasks such as walking, squatting, bending, standing, sitting, and rising from bed while wearing IMU sensors. Then, FGA was used to categorize individuals into the high and low fall risk group. Then a feature selection is applied to the data. During the classification process, multiple ML classifiers were used, and a CNN-LSTM hybrid model was created to identify the gait pattern from kinematic data in order to feed it into the model. Overall, all models showed positive outcomes, with RF surpassing others with a 91.3% accuracy score.

In 2023, a study was undertaken to identify older persons who are at high risk of falling using machine learning and multifactor analysis (Lyu et al., 2022). The dataset utilized 126 sets of motion trajectories from 42 markers in the sagittal, coronal, and transverse planes. These trajectories were gathered from 46 subjects in Beijing, all of whom were aged 60 or older. The process involves using a Wilcoxon rank sum test to determine any disparities between their age and BMI. The subjects were categorized into two groups: fallers and non-fallers. Fall was identified based on the codes MB46.3 and MB47.C from the ICD-11. The subjects participated in TUG tests in which 15 cameras and a calibration model recorded their motion trajectory. The final results indicated that the instability of the faller group was considerably higher than that of the no-faller group in both the male and female cohorts (*p* < 0.005). This emphasizes the importance of hip joint position in influencing human falls. Furthermore, the GBDT classifier surpasses the others with a perfect accuracy score of 100%.

The study (Kausar et al., 2023) proposed an approach that utilizes ADLS data and a wearable fall detection device to differentiate falls in elderly individuals. The study employed the SisFall dataset, a publicly available dataset consisting of 4,510 15-s signal segments, containing 1,789 falls and 2,707 ADLs. The study utilized unique processing techniques and feature extraction methods to extract features from accelerometry data. The method of feature extraction relied on a time window that moved continuously. Various window sizes have been examined to determine the optimal window size in terms of detection accuracy and computing efficiency. SVM, KNN, RF, and ANN are subsequently utilized for classification. The result demonstrates that SVM and RF exhibit outstanding performance with a 99% accuracy rate.

### 2.2 Deep learning methods

A One–One–One Deep Neural Networks algorithm was proposed for human fall risk prediction (Savadkoohi et al., 2021). The study used an open-source force-plate dataset to quantify human balance from a wide range of demographics of human participants as well as different standing situations, such as different surfaces and eye conditions. In the study, the proposed One–One–One Neural Networks classifier outperformed the other models by achieving a maximum accuracy of 99.9%. However, the model might have an issue with overfitting.

On the other hand, a group of researchers (Tunca et al., 2020) proposed a method for fall risk assessment with the use of inertial sensors. The dataset consisted of gait data that was collected by an IMU device from 90 elderly patients who were suffering from different neurological illnesses. The patients were separated into two groups depending on their history of falls, including high-risk and low-risk subjects. The study utilized different classifiers for fall risk detection, including LSTM, MLP, RF, HMM, and SVM. The overall accuracy was promising, while LSTM outperformed the other classifiers by achieving an accuracy of 92.1% on a separate test dataset collected from 16 patients. However, a limited dataset was deployed in this study, which might affect the model generalization.

A Deep Learning Enabled Fall Detection (DLFD) approach (Anwary et al., 2022) was introduced with the use of gait analysis. The method consisted of several steps, including data acquisition, edge communication, fall detection, gait extraction from pose estimation, post-processing, and normalization, fall and no-fall labeling, and model evaluation. The method deployed the pose estimation (MediaPipe) framework to extract gait information from real-time video taken by cameras positioned at different spots. After that, the retrieved gait data were processed, normalized, and labeled in preparation for bi-LSTM model training and testing. Lastly, the performance of the LSTM model achieved an impressive result for classifying falls and no-falls, with an accuracy of 96.35%, precision of 95.21%, and recall of 95.08%. However, the small number of vision-based real-life fall video datasets made it difficult to evaluate and train the DLFD approach.

Besides, a group of researchers (Choi et al., 2022) proposed a deep learning-based near-fall detection algorithm for a fall risk monitoring system. The algorithm used in the study was a modified directed acyclic graph convolutional neural network (DAG-CNN) architecture that extracted multi-level features from the input data. The dataset was collected with a single IMU device. The data were collected from 34 young volunteers (21 males and 13 females) with a range of ages 21 to 34, body masses ranging from 45 to 81 kg, and heights ranging from 1.57 to 1.85 m. There were 36 different categories of activities, including 10 different types of falls, 10 different types of near-falls, and 16 different types of near-falls. In the study, the dataset was split into training and testing sets, and a leave-one-participant-out cross-validation technique was applied for evaluation. Graph convolutional neural networks (DAG-CNN) and CNN were used in the classification process. Overall, the performance results obtained were favorable, while DAG-CNN had the best result with an accuracy of more than 98%. The weakness of this research is that the fall simulation experiment was carried out in a laboratory setting, which may not exactly represent actual falls.

The use of a CNN-RNN architecture-based approach (Philip et al., 2023) was proposed for fall detection. The experiment involved several steps, including data collection, data preprocessing, machine training, and performance evaluation. The study used ADls (daily living activities) datasets that contained gyroscope and acceleration data from 20 people doing various ADLs and simulating falls. In the preprocessing phase, the data was divided into fixed-length windows and normalized to have a mean and variance of zero. Furthermore, data augmentation techniques were also applied to account for changes in sensor positioning, such as introducing random noise and shifting the data. After that, the dataset was split into a ratio of 70:15:15 training, validation, and testing sets. In the training phase, the CNN module was trained to learn spatial features from the acceleration and gyroscope data, while the RNN module learned temporal dependencies and long-term trends. Overall, the performance was excellent, with an accuracy of 95%, precision of 94%, recall of 96%, an F1-score of 95%, and an AUC-ROC of 0.98. However, the limited dataset may affect the model generalization. The model was built and evaluated using only simulated falls, it might not accurately represent the complexity and diversity of actual falls. A summary of the state-of-the-art methods is presented in Table 1.

**Table 1:** Summary of related work.

Authors	Methodology used	Dataset and input details	Performance	Pros	Cons
Chakraborty and Sorwar (2022)	AdaBoost and Decision Tree	The study used a publicly available dataset, the long-term movement monitoring dataset, which was gathered from 71 elderly individuals. The participants included both fallers and non-fallers.	AdaBoost: 76% Decision Tree: 96%	Use of real fall data: The methodology is tested using a dataset of long-term accelerometer-assisted natural fall data, which enhances the validity and applicability of the research findings.	The analysis reveals that certain features did not perform well in the fall risk classification. This suggests that further improvement in feature selection methods could enhance the overall classification performance. The dataset is limited to 71 older adults, which may not be representative of the entire population.

Beauchet et al. (2018)	MLP, Averaged Neural Network (avNNET), and NEAT	The dataset comprises 848 elderly inpatients and includes baseline characteristics of the participants, such as age, gender, daily drug intake, usage of psychoactive medicines or analgesics, history of previous falls, and several measures of mobility and cognitive function.	MLP: 81.44% avNNET: 17.86% NEAT: 83.83	The study utilized a prospective cohort design. Used a hard outcome, represented by the occurrence of falls, which adds to the reliability and validity of the findings. Use of sophisticated statistical models.	The study only included individuals from one center, which may have limited the generalization of the results to other contexts or demographics. Limited ability to identify high-risk inpatients. A high rate of missing data, which could lead to bias and impair the representativeness of the results.
Velusamy et al. (2023)	DT, SVM, Neural Networks, RF, and Logistic Regression	Several public datasets were used in this study, including FARSEEING, UP-Fall, WISDM, UVA-Fall, and MobiAct datasets.	The accuracy of fall prediction ranges from 84.9 to 98.5%.	Real-time prediction. Combine information from a different sensor, enable full gait analysis.	Limited sample sizes. Sensor placement and reliability.
Aqueveque et al. (2022)	RF and SVM	The dataset utilized consists of mobility data from 71 elderly people, with two being excluded after feature extraction, resulting in a total of 69 subjects. Among these subjects, 31 experienced falls whereas 38 did not.	RF Accuracy: 81–98% F1-score: 70% SVM Accuracy: 59% Recall: 91% F1-score: 71%	The results showed promising potential for identifying subjects at risk of falling.	Limited dataset size. The use of an external database did not have any control over the data that the original researchers had provided regarding the classification methodology.
Ta and Jin (2023)	Logistic Regression, KNN, RF, SVM, and K-Mean Cluster	The dataset comprises 354 samples gathered from 23 patients with neurological disorders and balance impairments, as well as 262 patient datasets provided by qualified physiotherapists. The 262 patients with a variety of medical conditions are all at risk of falling and have a BBS score.	LR: 0.965421 KNN: 0.96215 RF: 0.963551 SVM: 0.965888 K-Mean Cluster: 0.90678	Comparative evaluation. High accuracy.	Limited dataset, which leads to bias.
Ullrich et al. (2023)	SVM, RF, and Gradient Boosting Classifiers	The dataset consisted of 422 days of sensor recordings, including 1,059 4x10MWTs, 55,965 real-world walking bouts, and 1,444,985 parameterized strides, collected from 40 patients with idiopathic PD who participated in the FallRiskPD study between March 2019 and June 2021.	RF Accuracy: 74% Sensitivity: 60% Specificity: 88% SVM-rbf Accuracy: 64% Sensitivity: 60% Specificity: 68% SVM-linear Accuracy: 57% Sensitivity: 50% Specificity: 64%	The study used long-term real-world data, which has advantages over supervised data.	The study’s unsupervised nature results in several limitations, including the varying amount of data provided by participants, differences in the number of recordings and gait test executions per day, and missing recordings due to technical and usability difficulties encountered by participants when handling the recording.
Lockhart et al. (2021)	RF	The dataset involves 171 community-dwelling older adults who participated in a 10-meter walking test while wearing an IMU sensor, which has a total of 58 parameters, including linear and nonlinear gait parameters.	RF: 81.6%	The study provides a useful method for assessing and quantifying gait abnormalities in fall-prone individuals, especially older adults. It emphasizes the importance of linear and nonlinear gait variables in identifying gait impairments and fall risk.	The limited generalizability of the model is due to the lack of patient-specific training data sets at the beginning of the data collection phase, which affects the model’s applicability to different populations.
Savadkoohi et al. (2021)	CNN, RNN, LSTM, One–One-Three, One–One-Two NN, and Proposed One–One–One NN	Open-source force-plate dataset that quantified human balance from a wide demographic of human participants (163 females and males aged 18–86) for varied standing conditions (eyes-open firm surface, eyes-closed firm surface, eyes-open, foam surface, eyes-closed foam surface) was used. It also used the Falls Efficacy Scale (FES), International Physical Activity Questionnaire (IPAQ), and Trail Making Test (TMT).	CNN: 99.3% RNN: 96.9% LSTM: 98.3% One–One-Three NN: 99.5% One–One-Two NN: 99.7% One–One–One NN: 99.9%	One–One–One Neural Networks classifier has a simple architecture, eliminating the need for a more complex model. Capability to extract the maximum amount of required spatiotemporal information from the force-plate using randomized sample datasets.	Due to the problem of overfitting, the model’s generalizability may be limited.
Tunca et al. (2020)	Proposed method: LSTM Traditional method: MLP, RF, HMM, and SVM	The dataset contains gait data from 90 subjects (patients) suffering from various neurological illnesses, such as Parkinson’s disease, vascular dementia, frontotemporal dementia, dementia with lewy bodies, and normal pressure hydrocephalus. The inclusion criteria for the individuals were the presence of a neurological disorder with gait-related symptoms and the ability to walk unassisted for at least 10 strides.	SVM Accuracy: 0.833 AUC: 0.894 RF Accuracy: 0.843 AUC: 0.894 MLP Accuracy: 0.903 AUC: 0.958 HMM Accuracy: 0.866 AUC: 0.957 LSTM Accuracy: 0.921 AUC: 0.957	The study employed various classifiers, including SVM, RF, MLP, HMM, and LSTM, for comparison. This approach allows for a comprehensive evaluation of the performance and effectiveness of different classification methods in fall risk assessment. The LSTM classifier showed a consistent performance with minimal fold-wise change, implying its dependability in assessing fall risk.	Limited availability of data.
Anwary et al. (2022)	LSTM	The study utilized three publicly accessible datasets, including the Fall Detection Dataset (FDD), the MMU Fall Detection Dataset, and the Multiple Camera Fall (MCF).	Accuracy: 96.35% Precision: 95.21% Recall: 95.08%	Accurate fall detection.	A small number of vision-based real-life fall video datasets makes it difficult to evaluate and train the DLFD approach. This restriction may limit the method’s ability to generalize in practical situations.
Choi et al. (2022)	DAG-CNN and CNN	The dataset used in the research consists of 1,200 samples collected from 20 participants, which include both near-fall and non-fall activities of daily living (ADLs).	DAG-CNN: 98% CNN: 69.8 and 89.7%	High predictive accuracy.	The fall simulation trials were carried out using a laboratory scenario that may not fully represent actual falls.
Philip et al. (2023)	CNN-RNN	The dataset utilized a publicly available dataset consisting of instances of falls and everyday activities. The activities of daily living (ADLs) are recorded by a wearable sensor device, comprising gyroscope and acceleration data collected from 20 individuals performing various ADLs and simulating falls.	Accuracy: 95% Precision: 94%, Recall: 96% F1-score: 95% AUC-ROC: 0.98	Accurate and dependable.	Limited dataset size: The number of people and falls in the dataset used for training and testing the model is constrained, which may limit the broad applicability of the findings. Due to the model being built and evaluated using only simulated falls, it might not accurately represent the complexity and diversity of actual falls. Lack of consideration for contextual factors: The model did not consider the context and environmental aspects that can affect fall risk and detection.
Angsuwan et al. (2024)	RF and Naïve Bayes	The dataset consists of 160 accelerometer gait data and demographic data. Each participant involves wearing the device and performing the TUG assessment twice.	AUC score RF: 0.98 NB: 0.8	The effectiveness of Random Forest in fall risk prediction.	Limited dataset size.
Nishiyama et al. (2024)	GBDT	The dataset contains acceleration and rotational velocity data from an IMU acquired from 44 elderly people (22 fallers) using a smartphone to record their process of walking for 6 s under a 10-meter walking path four times.	GBDT: 0.936	The novel method requires only a single gait cycle.	Constraints preventing frequent or long-distance walking or for usage in settings with limited walking areas.
Kou et al. (2024)	SVM, LR, RF, KNN, and NB	The data includes 28 kinematic data from 28 workers aged 60 to 80 who wore IMU sensors while doing various tasks like walking, squatting, bending, standing, sitting, and rising from bed.	SVM: 87.5% RF: 91.3% LR: 86.96% KNN: 87.5% NB: 85.71%	The effectiveness of the ML approach and the use of a single IMU.	The small dataset size affects the model’s generalizability.
Lyu et al. (2022)	L1/2 sparse iteration, SVM, GBDT, RF, DNN, and RNN	The dataset included 126 sets of motion trajectories from 42 markers in the sagittal, coronal, and transverse planes, collected from 46 subjects aged 60 or older.	L1/2 sparse iteration: 60.87% SVM: 97.83% GBDT: 100% RF: 93.48% DNN: 56.52% RNN: 19.57%	The effectiveness of multifractal algorithms and machine learning approaches.	The small size of the dataset, which may result in model overfitting.
Kausar et al. (2023)	SVM, KNN, RF, and ANN	The SisFall dataset comprises 1,789 instances of falls and 2,707 ADLs collected from a group of 23 young adults aged between 19 and 30. The dataset contains 15 different kinds of falls, including falls while walking, falls forward, and falls backward while sitting. Furthermore, it has 19 labeled ADLs, such as walking upstairs and downstairs, as well as walking and jogging.	SVM: 99.75% RF: 99.73% KNN: 96.34% ANN: 99.05%	The efficacy and cost-effectiveness of the proposal, which can attain promising outcomes.	The dataset exhibits inadequate diversity since it only includes a young age demographic.

## 3 Proposed solution

**Figure 1:**
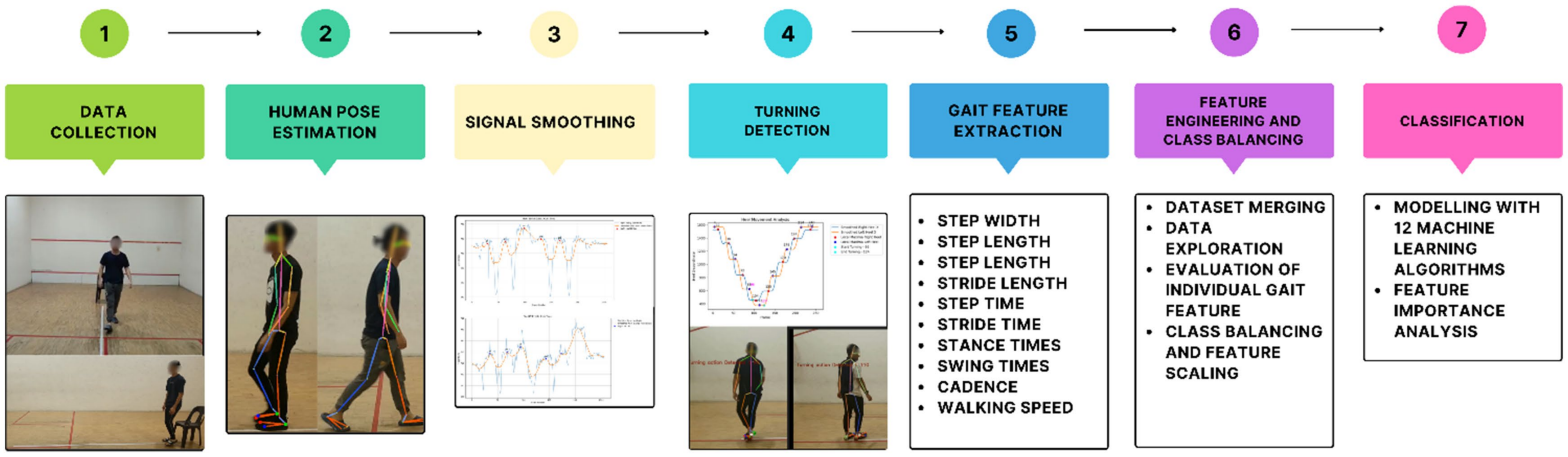
System workflow: gait analysis and classification process.

This section presents the methods and system workflow used in this study. The procedure consists of seven primary steps depicted in Figure 1.

### 3.1 Dataset collection

**Figure 2:**
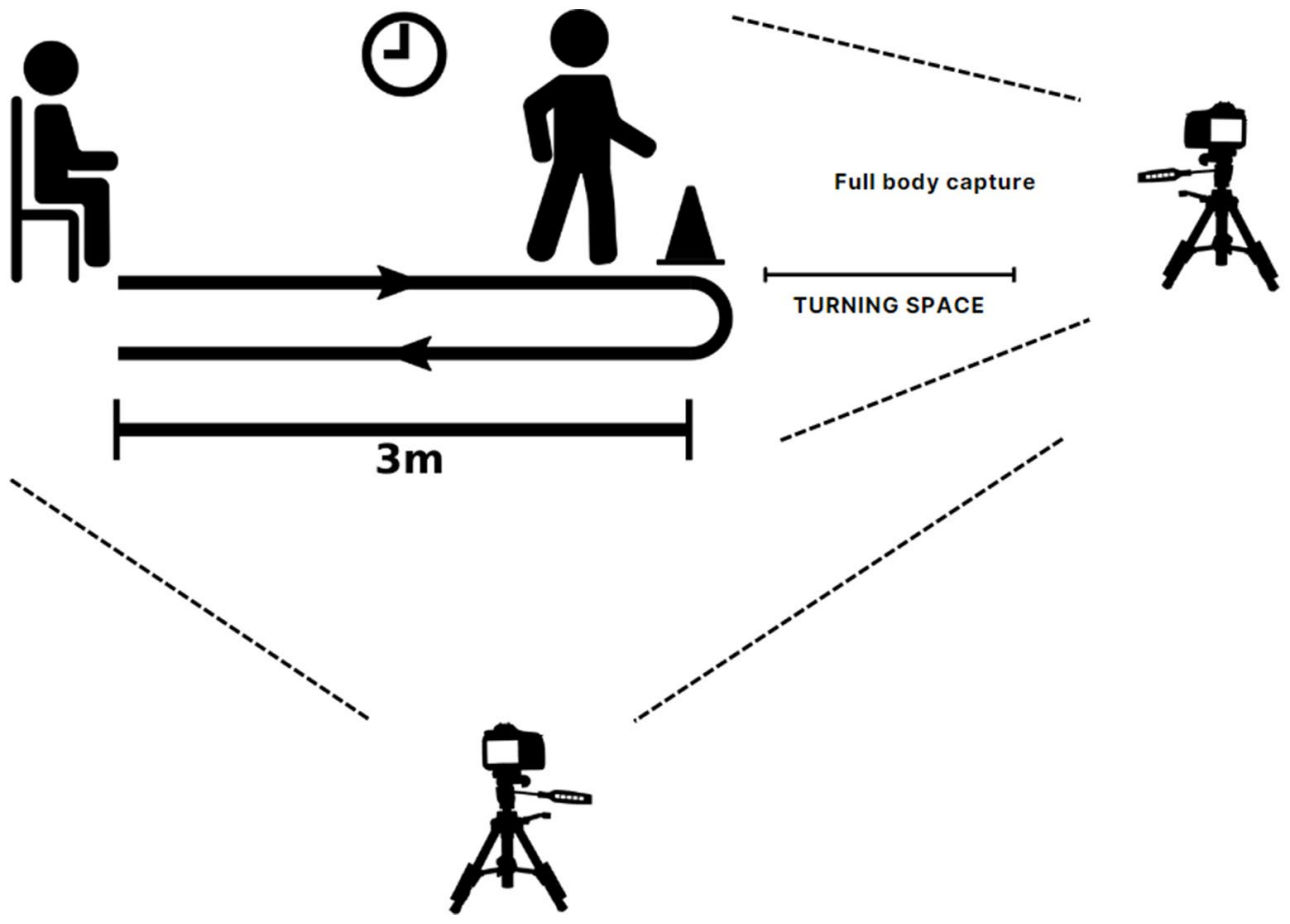
Data acquisition via TUG procedure.

This dataset comprises 25 subjects with varied gender, ethnicity, and age groups (4 females and 21 males). The age distribution includes 19 subjects ranging in age from 20 to 35, 2 subjects aged around 50 to 59, and 4 subjects aged 60 and above. Among these 25 subjects, 4 were fallers, as determined by the JHFRAT evaluation. They were requested to undergo the Time Up and Go tests, which involved starting from a seated position, walking for 3 meters at a normal pace, then returning to the chair and sitting down (refer to Figure 2) (Time Up and Go (TUG), 2024a). During the TUG assessment procedure, two cameras and tripods were set up to capture the front and side views at 30 frames per second, with a 16:9 ratio of 1080p video resolution. This study uses 21 non-faller samples from this dataset as a healthy control group for this research. The dataset is known as the MMU Fall Risk Prediction (MMU-FRiP) Dataset henceforth.

### 3.2 Human pose estimation

Human pose estimation is a computer vision approach that can track human poses in images and videos. AlphaPose (Fang et al., 2023) is one of the well-known frameworks known for its high performance in human pose estimation tasks in images and videos. It comes with several features like multi-person pose estimation, real-time performance, and multi-camera pose tracking. It can be used to analyze the image input or video input frame by frame and detect human body parts. In this study, it is employed to extract landmark body joint positions from the video input.

**Figure 3:**
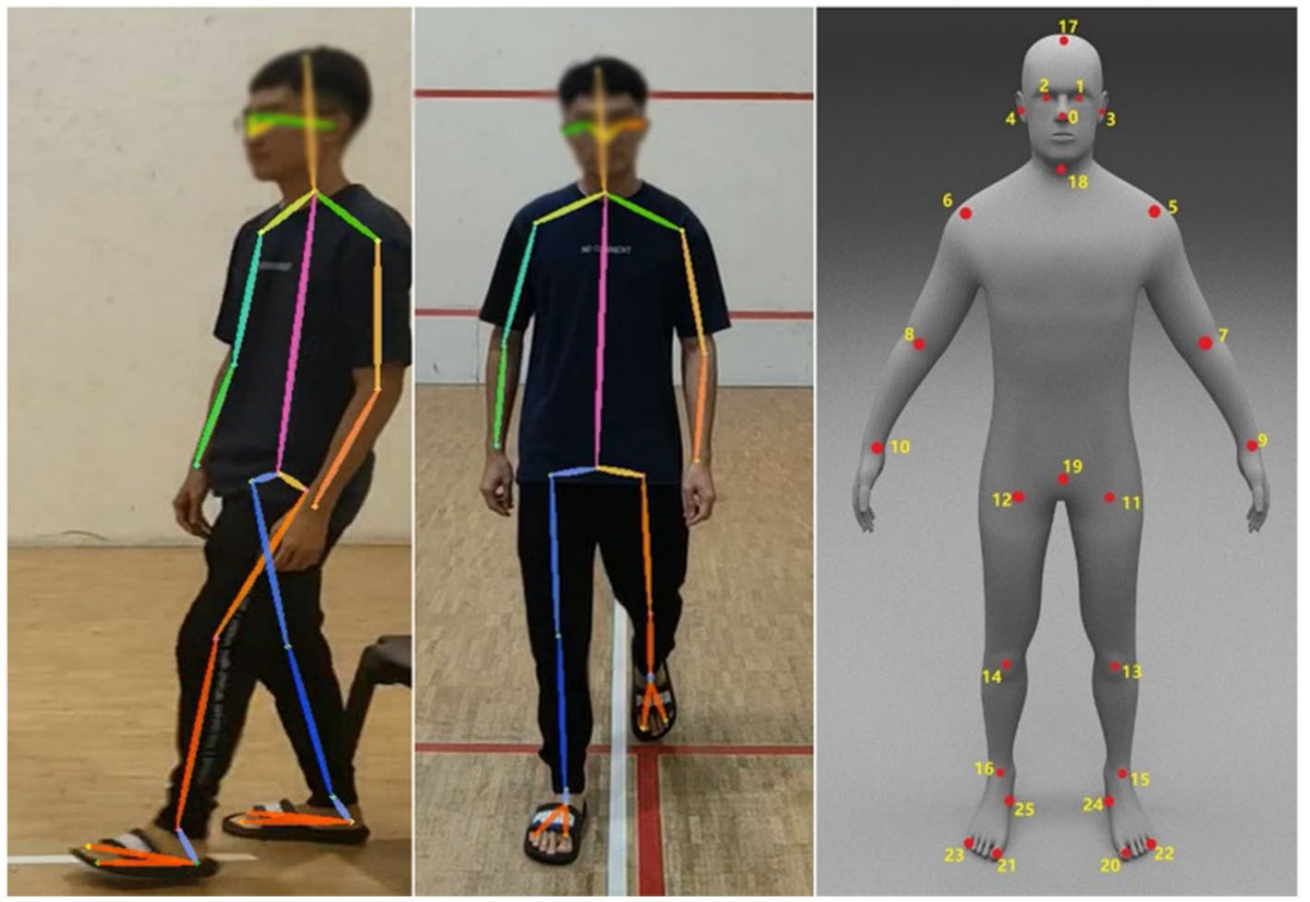
Halpe Full Body model output and keypoint illustration.

Halpe Full Body model is used as a posture-tracking model for video processing frame by frame. The generated output includes 26 key points, such as nose, left eye, right eye, left ear, right ear, left shoulder, right shoulder, left elbow, right elbow, left wrist, right wrist, left hip, right hip, left knee, right knee, left ankle, right ankle, head, neck, hip, left big toe, right big toe, left small toe, right small toe, left heel, and right heel. The extracted body key points are shown in Figure 3.

### 3.3 Signal smoothing

Savitzky–Golay (SG) filter is a popular signal processing technique for reducing noise and improving signal smoothness (Pelliccia, 2019). The concept of SG smoothing is straightforward. The SG algorithm first selects a window around each data point in the spectrum, then fits a polynomial to the points in the selected window, and finally replaces the data point in question with the appropriate value of the fitted polynomial (Pelliccia, 2019). The user can specify the window size and the order of the polynomial to be fitted to the input. The window size refers to the filter window’s length, which corresponds to the number of coefficients. The polynomial parameters refer to the order of the polynomial used for fitting the samples into the window. This order affects the degree of the polynomial used in the smoothing process. The formula to obtain the smoothed data is presented below:


(1) y^i=∑j=−kkcj..yi+j

-
y^i represents the smoothed value at the i index position in the data series.
-
$c_{j}$ referring to the filter coefficients
-
$y_{i+j}$ are the data points in the neighborhood of the central point 𝑖 (Pelliccia, 2019).

**Figure 4:**
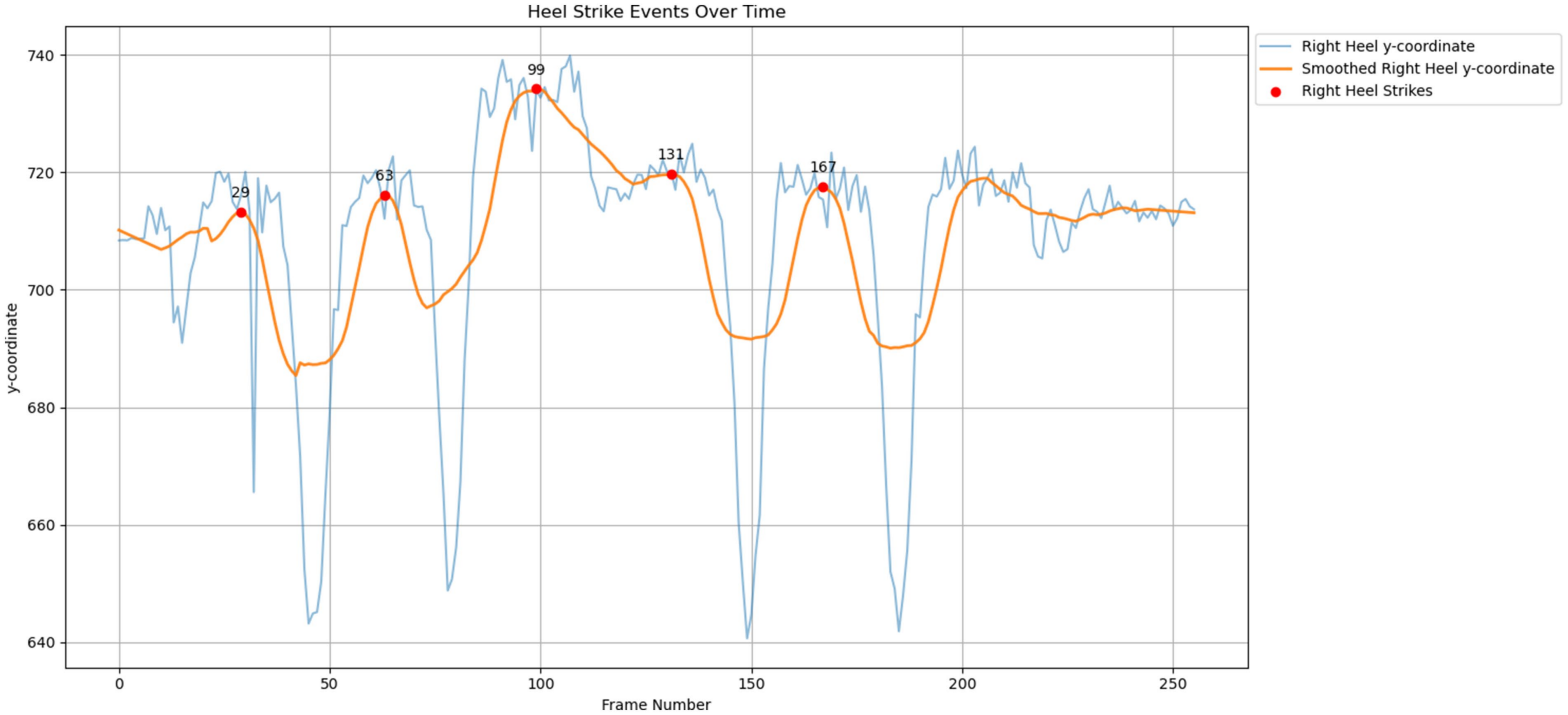
Signal smoothing on heel strikes events over time.

In this study, the SG filter was employed to smoothen the heel strikes and toe-offs signals based on the *y*-coordinate of frames, shown in Figure 4, respectively. The reason for using the *y*-coordinate for the heel and toe frames is that walking involves the vertical motion of lifting and lowering the feet. Therefore, using vertical frames provides more accurate results and comprehensible visualization for heel strike and toe-off detection. The heel strikes and toe-offs locations are required to calculate the relevant gait features.

### 3.4 Turning detection

The goal of recognizing the turning frames is to prevent it from being included in gait feature extraction. The rationale behind this is that the turning action forces the participants to change their movement, which is not their normal walking pace. As a result, gait events detected during turning actions such as heel strikes, and toe offs may result in incorrect data within the gait feature extraction process. Therefore, frames containing turning motion are excluded to ensure the integrity of gait analysis.

Turning detection involves several logics and steps. To identify turning, we find the lowest value from the local maxima observed in the data. The underlying idea is that the deepest points identified on the right and left feet correspond to moments where the subject momentarily stops to adjust both feet to facilitate the turning process. After that, a comparison is performed to ascertain which foot exhibits the deepest and the second deepest points. The rationale is that the deepest point is likely to indicate that the next point will be the initial heel strikes following the completion of the turning motion. The second deepest point might indicate the final heel strike before the turn. With this, we can determine the last heel strike before the turn and the first heel strike after the turn. A median operation is performed to calculate the starting and ending frames of the turning movement. The steps for turning frames detection are summarized as follows:

1. Local Maxima Identification: Detect local maxima within the data to locate the heel strikes events, i.e., local_maxima_r and local_maxima_r. During the process of identifying local maxima, the Savitzky–Golay filtering parameters are set at 11 window lengths and 3 polynomial orders. These parameter values were chosen because they generate a generalized output that can be used to different age groups’ toe off and heel strike signals. The determination is evident in the individual keypoints’ signal evaluation through visualization and video analysis. On the other hand, the parameter min_distance in the find peak function is used to determine the minimum distance between neighboring peaks in the peak detection algorithm.
2. Deepest Points Determination: Identify the deepest points (lowest value) among the local maxima for both heels. These points are likely corresponded to instances within the turning motion, signifying moments where the path requires a directional change, thereby indicating the last heel strikes in a turning position, i.e., *deepest_maxima_r_index* and *deepest_maxima_l_index*.
3. Heel Strike Frame Analysis: After acquiring the deepest points from both heels, the next steps involve distinguishing the frames indicative of the starting and ending of the turn. Toward this end, the deepest points are compared to find the deepest and second deepest points, i.e., *deepest_index* and *deepest_index2*. The frame preceding the deepest point is considered the last heel strike before turning. Conversely, the frame following the second deepest point is regarded as the subsequent heel strike post-turn. Before moving on to the next phase, we must determine whether *deepest_index* and *deepest_index2* belong to the left or right feet, i.e., *first_deepest_is_in_r* or *first_deepest_is_in_l* and *second_deepest_is_in_r* or *second_deepest_is_in_l*. This can be achieved by determining whether the index is found in *local_maxima_r* or *local_maxima_r*. After that, we can obtain the *index_before_deepest* and *index_after_deepest*. The *index_before_deepest* relates to the last heel strike before turning, which is the index of local maxima before *deepest_index2*. While *index_after_deepest* relates to the initial heel strike after the turn, it is the index of local maxima after *deepest_index*.
4. Median Calculation for Turning Frames: Median operation is performed to find frames marking the beginning and end of the turn:

(2) $$\begin{array}{l} \mathrm{star}t_{\mathrm{turning}}=\mathrm{Median}\left(\mathrm{inde}x_{\mathrm{befor}e_{\mathrm{deepest}}},\;\mathrm{deepe}s_{\mathrm{index}2}\right),\;\mathrm{end}\_\mathrm{turning}\\ \;=\mathrm{Median}\left(\mathrm{index}\_\mathrm{after}\_\mathrm{deepest},\;\mathrm{deepest}\_\mathrm{index}\right) \end{array}$$

The processes involved in finding turning frames are summarized in Algorithm 1.

#### ALGORITHM 1: Pseudocode for Finding Turning Frames


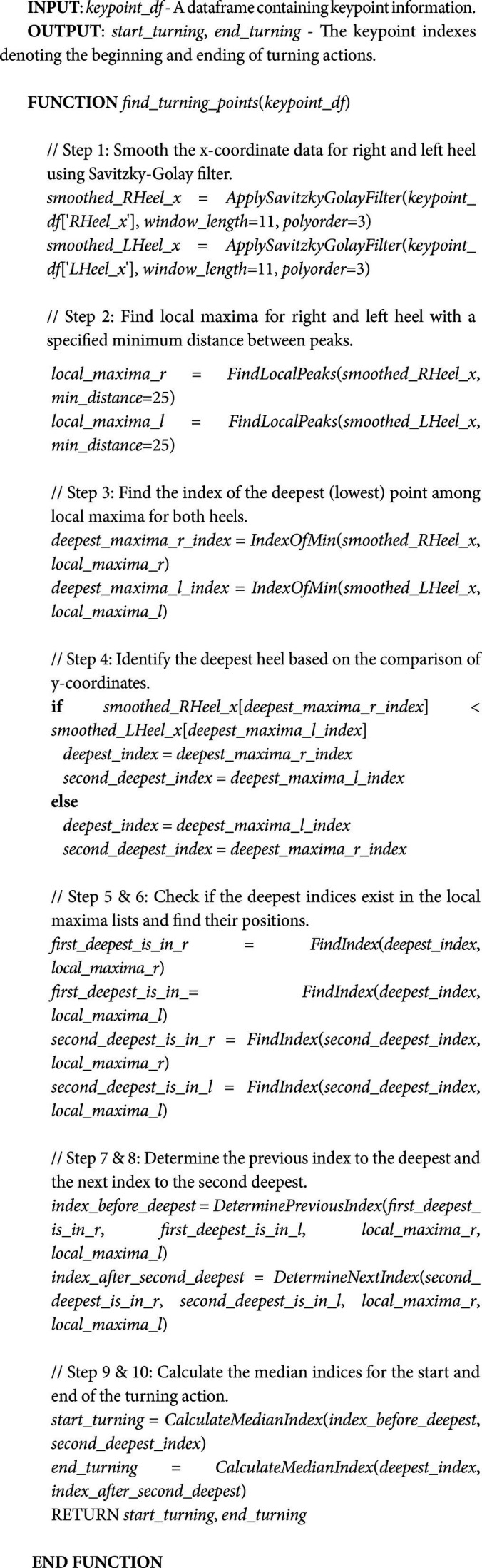


### 3.5 Gait feature extraction

This study focuses on the analysis of two specific key points, namely the Heel and the Big Toe. These key points are utilized to identify frames and detect relevant events. Table 2 provides some explanation for the heel strikes and toe-off features.

**Table 2:** Heel strikes and toe offs details.

Gait events	Description	Identification
Heel strikes $\left(\mathrm{Heel}\;\mathrm{Strike}\right)$	Heel strikes occur when the foot makes contact with the ground after the swing phase is over	Local Maximum of (LHeel&RHeel ) in y-coordinates
Toe offs $\left(\mathrm{Toe}\;\mathrm{off}\right)$	Toe offs occur when the toe begins to leave the ground.	Local Maximum of ( LBigToe&RBigToe ) in y-coordinates

Building upon features extraction, the formula and frame index that reflect the occurrence of heel strike and toe-off are used to calculate gait features such as stride length, step length, stance time, swing time, step times, stride time, cadence, and speed.

#### 3.5.1 Stance time

Stance time ( $\mathrm{Stanc}e_{T})$ refers to the time one foot spends on the ground during a gait cycle. The formula used for the toe off ( Toeoff ) events subtracted with heel strike ( HeelStrike) and divides with the frame per second ( FPS) to obtain the duration of stance.


(3) $$\mathrm{Stanc}e_{T}=\frac{\left(\mathrm{Toe}\;\mathrm{off}-\mathrm{Heel}\;\mathrm{Strike}\right)}{FPS}$$

#### 3.5.2 Stride time

Stride time ( $\mathrm{Strid}e_{T}$ ) refers to the time it takes to complete a stride. The formula is the next heel strike ( $\mathrm{Heel}\;\mathrm{Strik}e_{i+1})$ minus with current heel strike ( $\mathrm{Heel}\;\mathrm{Strik}e_{i})$ and dividing with fps ( FPS) .


(4) $$\mathrm{Strid}e_{T}=\frac{\mathrm{Heel}\;\mathrm{Strik}e_{i+1}-\mathrm{Heel}\;\mathrm{Strik}e_{i}}{FPS}$$

#### 3.5.3 Step time

Step time ( $Step_{T}$ ) refers to the time it takes to complete a step. The formula is the opposite of the toe off ( $\mathrm{Opposite}\;\mathrm{Toe}\;\mathrm{Of}f_{i}$ ) minus with heel strike ( $\mathrm{Heel}\;\mathrm{Strik}e_{i}$ ) divide with fps ( FPS) .


(5) $$Step_{T}=\frac{\mathrm{Opposite}\;\mathrm{Toe}\;\mathrm{Of}f_{i}-\mathrm{Heel}\;\mathrm{Strik}e_{i}}{FPS}$$

#### 3.5.4 Cadence

Cadence ( Cadence ) refers to how many steps you take in a minute. The formula is the total step ( Numberofstep) divided by the total duration in minutes [ $\mathrm{Total}\;\mathrm{duration}\;\left(\mathrm{minute}\right)$ ].


(6) $$\mathrm{Cadence}=\frac{\mathrm{Number}\;\mathrm{of}\;\mathrm{step}}{\mathrm{Total}\;\mathrm{duration}\;\left(\mathrm{minute}\right)}$$

### 3.6 Feature engineering and class balancing

This section covers the methods that help to enhance the model performance in classification tasks, which involve class balancing and feature engineering.

Imbalanced datasets can result in biased predictions and inaccurate accuracy. To resolve this issue, the solutions would be undersampling and oversampling. Undersampling is the process of reducing the number of instances in the majority class to achieve a balanced dataset. Nevertheless, this may not be suitable for tiny datasets. Oversampling is another method, but random oversampling might result in overfitting due to redundant data. SMOTE oversampling, which stands for Synthetic Minority Oversampling Technique, is beneficial in this scenario. The SMOTES implementation processes are given as follows:

- Step 1: Identifying the feature vector and its nearest neighbors.
- Step 2: Find the difference between both of them.
- Step 3: Multiply the difference by a random number between 0 and 1.
- Step 4: Generate a novel synthetic instance by incorporating the random number into the feature vector.
- The process will be repeated until the desired balanced level is met.

Standard Scaler is a feature scaling approach that helps to standardize feature values, which can be helpful for continuous variables that have a high-ranking difference. Furthermore, feature scaling could speed up convergence and improve machine-learning model performance. The formula to scale the feature vectors in this study is given by:


(7) $$z=\frac{x-\mu }{s}$$

### 3.7 LightGBM

LightGBM, or Light Gradient Boosting Machine, is a gradient-boosting technology developed by Microsoft to enhance speed and efficiency (Ke et al., 2017). LightGBM is a gradient-boosting framework that prioritizes leaf-wise tree growth, leading to a reduced number of nodes in the tree. It is well-known for its rapid pace and effectiveness, making it well-suited for handling extensive datasets and complex feature spaces.

LightGBM constructs trees using a histogram-based approach to learning. It categorizes continuous features into bins and creates histograms to efficiently determine the best splits. The approach builds trees by selecting the leaf with the highest delta loss during the process, rather than constructing them level by level. It utilizes techniques such as Gradient-based One-Side Sampling (GOSS) and Exclusive Feature Bundling (EFB) to improve training speed (Ke et al., 2017).

LightGBM is rapid and scalable, making it appropriate for large datasets and numerous feature spaces. It effectively manages categorical characteristics, reducing the need for one-hot encoding. This method is more memory-efficient and achieves higher accuracy than traditional gradient boosting methods. LightGBM may not be as effective with small datasets compared to other methods due to its less apparent efficiency improvements. Overfitting is more likely to occur when the dataset is limited. Complex models have less interpretability compared to simpler models.

## 4 Experiment results

### 4.1 Datasets

The experiments were performed using the 21 non-fallers group from MMU-FRiP dataset and Mendeley public datasets to predict fall risk. The reason for needing the public dataset is due to the challenges of recruiting participants with the risk of falling. The MMU-FRiP dataset used in this study consists of 21 samples, comprising 3 females and 18 males. Among these samples, 18 subjects are aged between 20 and 35, while 1 subject is aged around 50, and 2 participants are aged 60 and older. The Mendeley public datasets (Caicedo Rodríguez et al., 2020) contain 44 elderly individuals, averaging 69.98 years in age (standard deviation of 8.57 years), comprising 37 women and 7 men, assessed using the Performance-Oriented Mobility Assessment (POMA). In contrast, the public dataset was categorized under fallers, given that all included participants scored below 19 on the POMA scale, signifying a heightened fall risk.

In the process of merging the datasets, attribute mapping is required due to the limited number of features in the MMU-FRiP dataset and the incomparability of some feature values. Therefore, most spatial features were omitted, retaining primarily the temporal features for analysis. The following process involved two datasets put together to form a new dataset. The details are shown as follows:

- left_stride_time: Left_Stride_Time (Mendeley dataset) is aligned with left_stride_time (MMU-FRip dataset).
- right_stride_time: Right_Stride_Time (Mendeley dataset) is aligned with right_stride_time (MMU-FRiP dataset).
- left_step_time: Left_Step_Time (Mendeley dataset) is aligned with left_step_time (MMU-FRiP dataset).
- right_step_time: Right_Step_Time (Mendeley dataset) is aligned with right_step_time (MMU-FRiP dataset).
- left_stance_time: Left_Single_Support (Mendeley dataset) is aligned with left_stance_time (MMU-FRiP dataset).
- right_stance_time: Right_Single_Support (Mendeley dataset) is aligned with right_stance_time (MMU-FRiP dataset).
- Cadence: The average of Left_Cadence and Right_Cadence (Mendeley dataset) is aligned with cadence (MMU-FRiP dataset).
- The label is aligned with the label.

This study conducted two distinct experiments: (1) Experiment 1 used gait features separately for the right and left feet, and (2) Experiment 2 used the average of the right and left feet’s gait features. Experiment 2 was carried out to facilitate a more generalized understanding of gait dynamics, which may be more suitable for certain gait analysis contexts. Table 3 illustrates the dataset after attribute mapping, as well as the dataset that will be used for experiments 1 and 2.

**Table 3:** Dataset combinations.

Mendeley dataset	MMU-FRiP Dataset	Experiment 1 dataset	Experiment 2 dataset
Left_Stride_Time	left_stride_time	left_stride_time	average_stride_time
Right_Stride_Time	right_stride_time	right_stride_time	
Left_Step_Time	left_step_time	left_step_time	average_stride_time
Right_Step_Time	right_step_time	right_step_time	
Left_Single_Support	left_stance_time	left_stance_time	average_stance_time
Right_Single_Support	right_stance_time	right_stance_time	
Mean (Left_Cadence + Right_Cadence)	Cadence	Cadence	Cadence
Label	Label	Label	Label

### 4.2 Toe-offs, heel strikes, and turning detection analysis

**Figure 5:**
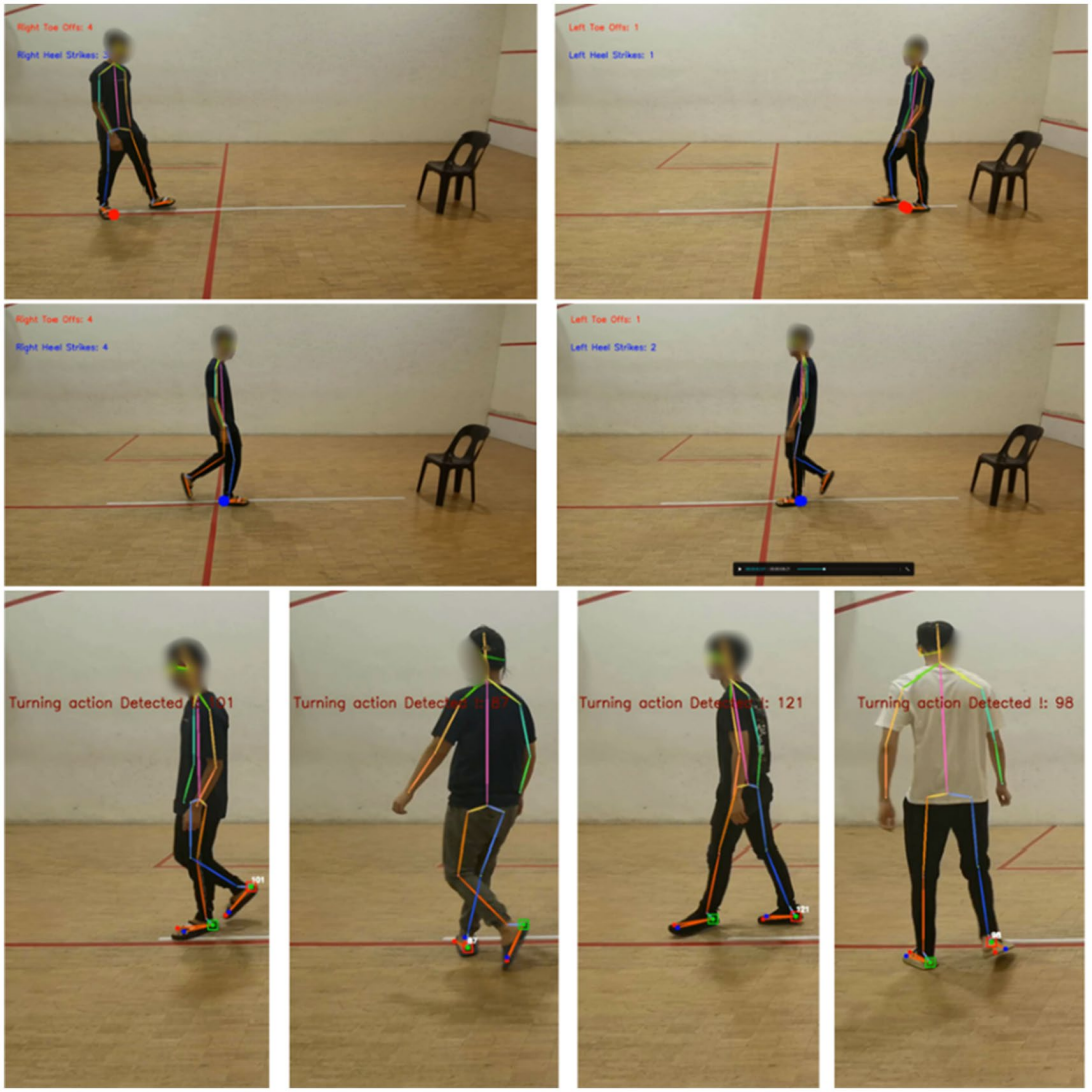
Toe offs, heel strike turning detection in gait analysis.

Firstly, an analysis is conducted to ensure that the gait events detection is working appropriately, to present the keypoint occurrence time as well as the signal to show that events occur. In Figure 5, the red dot represents the toe-off occurrence, and the blue dot indicates the heel strike event. The turning detection analysis is also carried out to remove the non-normally paced gait cycle, the square appears to highlight the foot when it is in the turning process, and the text also appears to display the turning frames. The following figure shows the event occurrence detection outcome, and the results are promising, indicating that the human pose estimation framework and signal smoothing work effectively.

### 4.3 Gait features analysis

**Figure 6:**
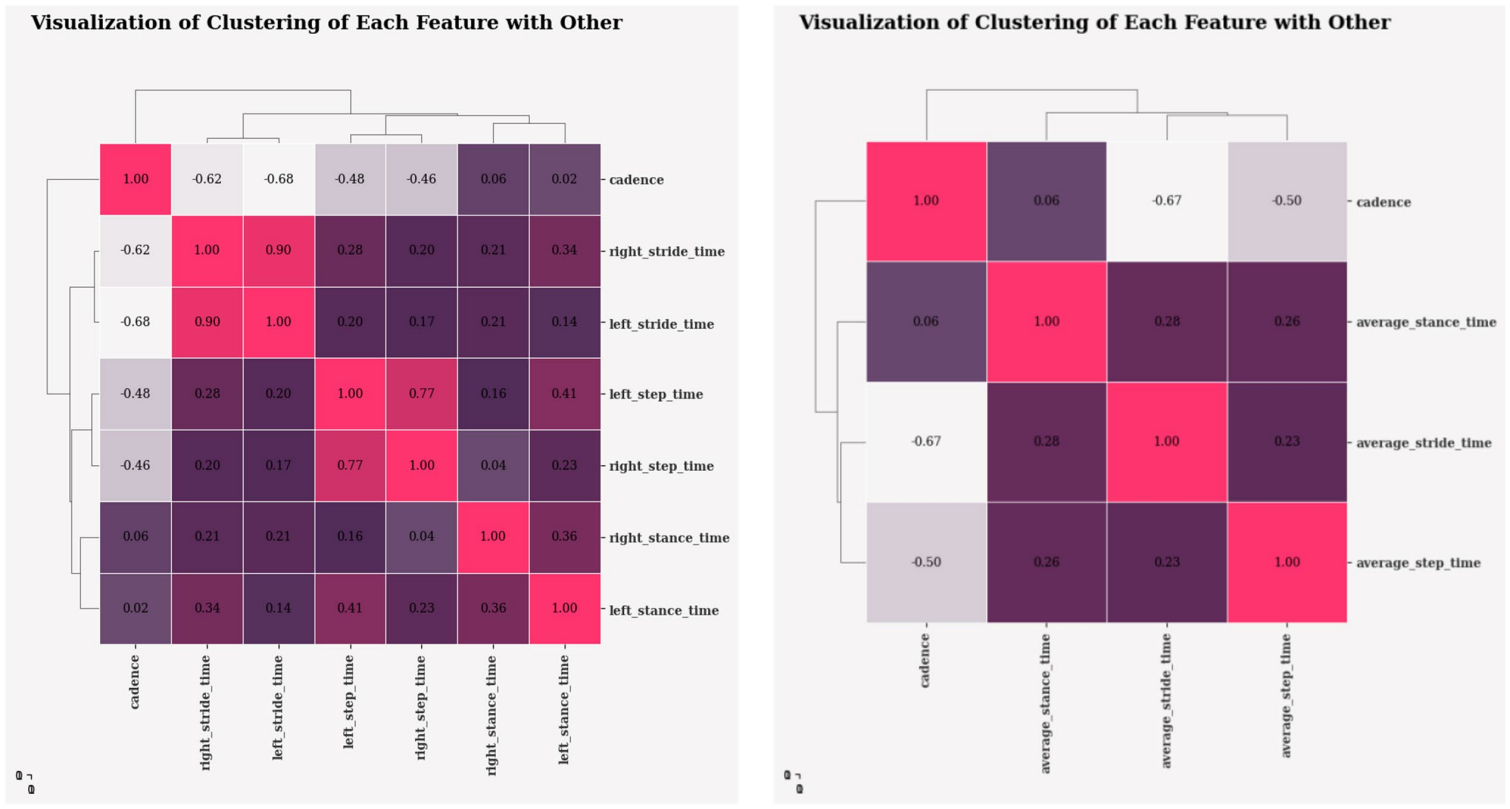
Dendrogram heatmap visualizing clustering of features based on correlation coefficients.

Figure 6 displays the extracted gait features and how they correlate with one another. The values in each cell show how the feature values are related to one another. Positive correlations indicate a positive relationship in which an increase in one feature value will lead to an increase in the other. Conversely, negative correlations show the opposite relationship, where an increase in one feature value would lead to a decrease in the other, and a 0 value denotes no correlation at all. Based on the observation, cadence appears to have a strong negative relationship with stride and step time, but a weak correlation with stance time. It indicates that when cadence increases, these negative correlation features tend to decrease, whereas low correlation may have no effect on the value, which appears to be usual because a quicker walking pace results in shorter step and stride durations. Stride time, stance time, and step time all correlated positively. Indicating an increase in one’s feature value may cause others to increase, which is common because longer strides require longer step and stance times.

### 4.4 Evaluation of individual gait feature

This section outlines each feature distribution, as well as the information that could be obtained from the analysis.

**Figure 7:**
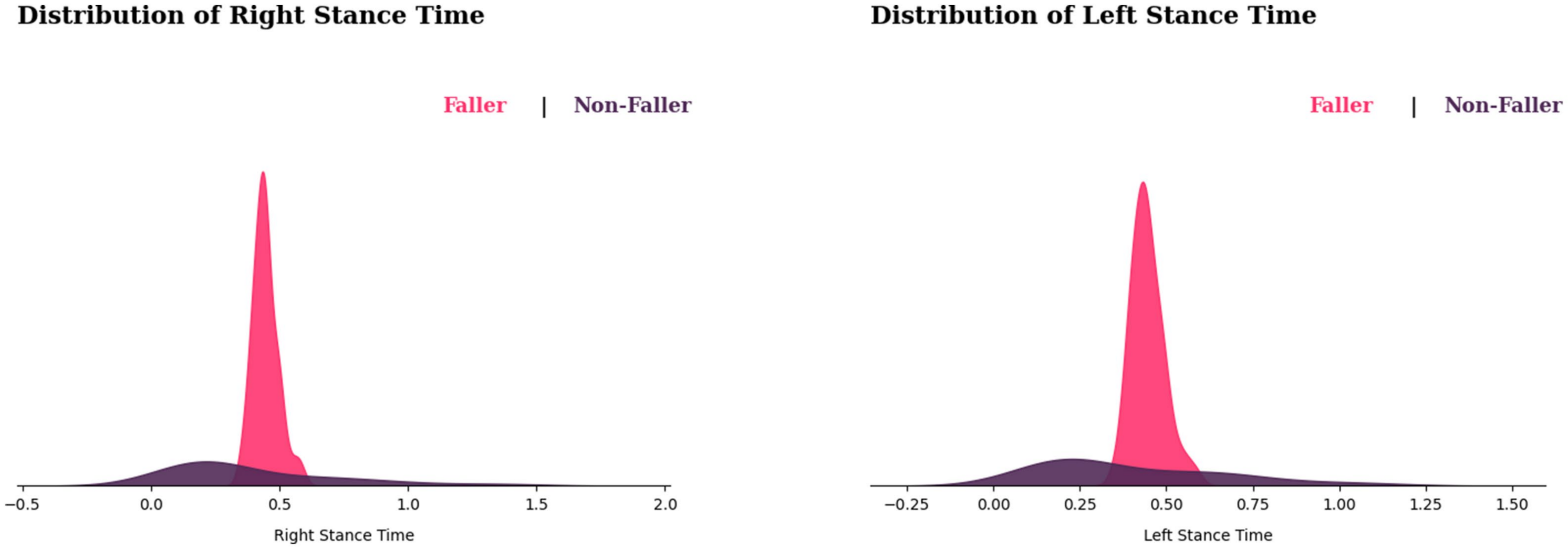
Distribution of stance times for fallers and non-fallers.

According to Figure 7, the distribution of stance time for fallers appears to be shifting to the right. This implies that fallers may have a greater stance time than non-fallers. However, there is some overlap between the fallers and non-fallers, therefore not everyone with a longer stance duration is more likely to fall, as some fallers may have a similar stance duration. In terms of variability, non-fallers appear to have a wider range of stance time variability, whereas fallers exhibit sharp peaks, indicating that they have a more consistent stance time.

**Figure 8:**
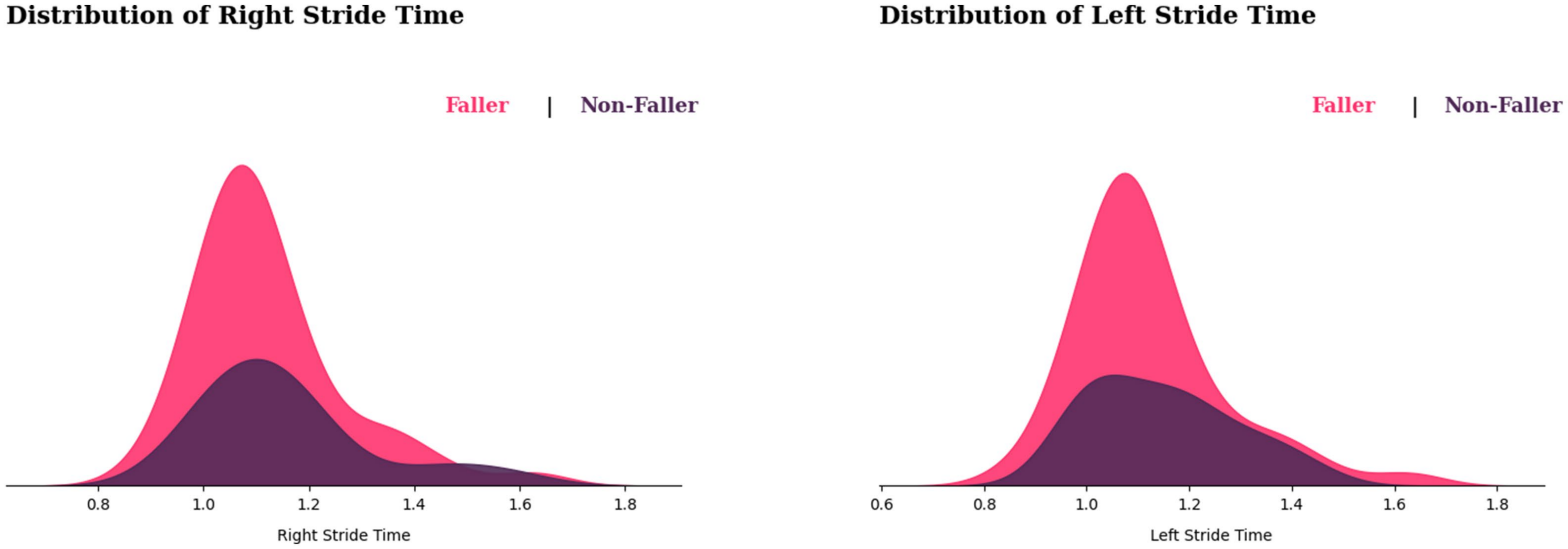
Distribution of stride times for fallers and non-fallers.

Based on Figure 8, the distribution of left and right stride times for fallers appears to be wider than for non-fallers. Furthermore, the fallers appear to be skewed to the left, suggesting that most fallers have shorter stride times. When it comes to observing differences in peaks in both distributions, the faller peaks around 1.1 on the left stride time, slightly right of the non-faller, while having similar peaks on the right side, indicating that the faller may have a slightly longer stride duration than the non-fallers. However, there is a significant overlap between the two distributions, implying that not all individuals with longer stride durations will fall. While, in variability comparison, fallers may have more variability in their stride time than non-fallers.

**Figure 9:**
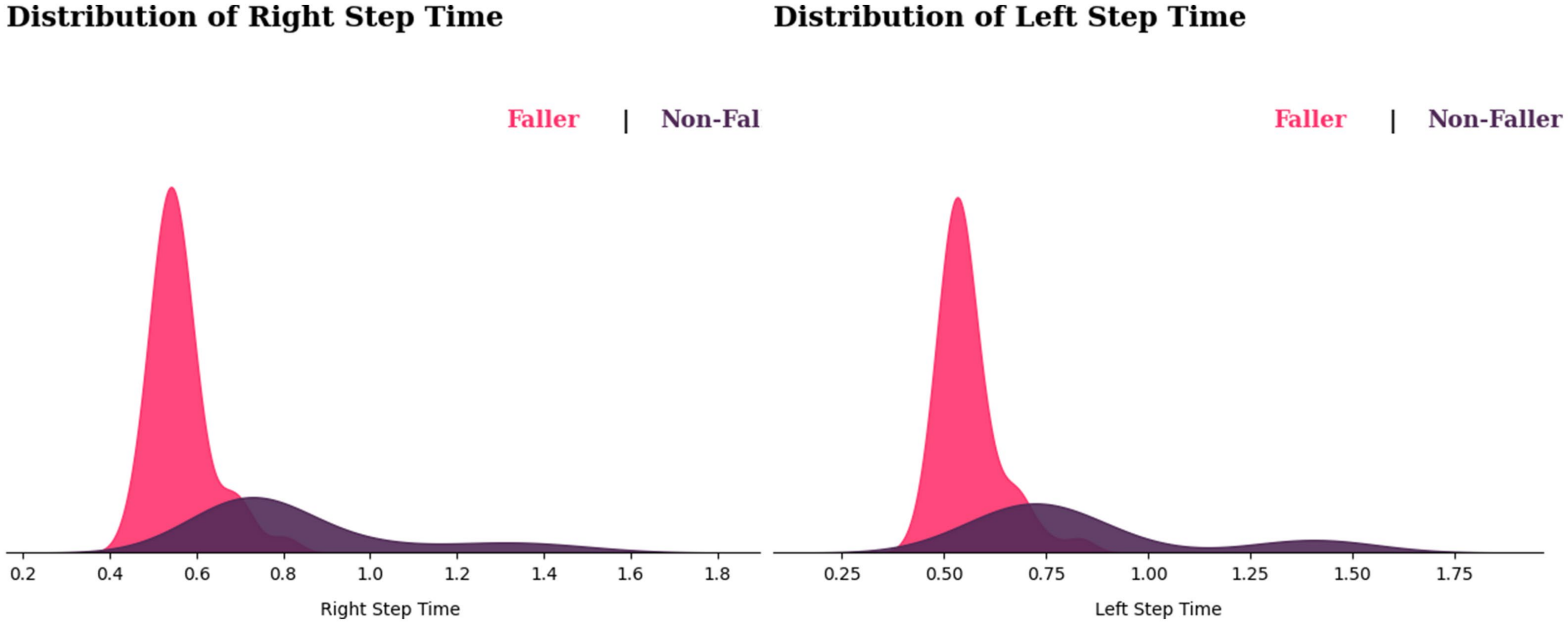
Distribution of step times for fallers and non-fallers.

We observe from Figure 9 that the fallers’ step time distribution appears to be shifted to the left side of the graph. This suggests that most fallers may have shorter step times than non-fallers. However, this does not determine that slower step time causes falls, because there is some overlap between the two distributions, implying that some non-fallers also have a shorter step time. In variability observations, non-fallers show a wide range of step time variability, whereas variable step time for fallers displays sharp peaks in their step time distribution, showing that those who fall most likely would have consistent step durations.

**Figure 10:**
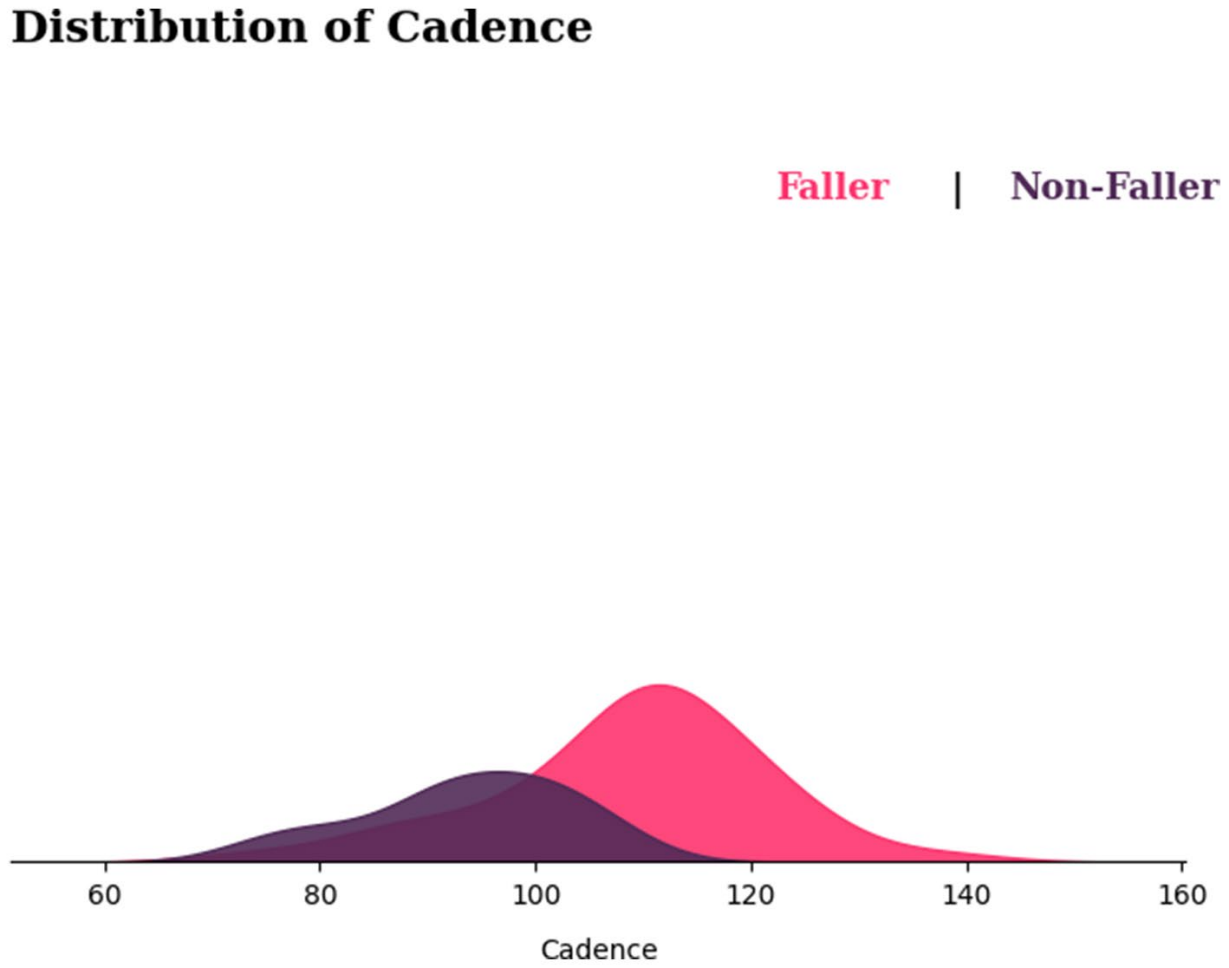
Distribution of cadence for fallers and non-fallers.

On the other hand, Figure 10 shows the cadence distribution for fallers and non-fallers, with fallers appearing to be shifted to the right side of the graph. This implies that fallers may have a higher cadence than non-fallers. Nonetheless, there is considerable overlap between the two distributions, suggesting that some fallers and non-fallers may have similar cadences. While in terms of variability, the fallers show greater variability in cadences compared with non-fallers.

### 4.5 Evaluating the effect of class balancing

This section presents the effectiveness of the SMOTE methods in handling imbalanced data. The dataset contains a total of 65 subjects, and 44 of them were fallers, indicating a serious class imbalance issue. The SMOTE resampling method is used to deal with the class imbalance problem.

Table 4 shows a significant improvement in all models after applying SMOTE in terms of model generalization. The noticeable impact of SMOTE demonstrates its efficacy in addressing the overfitting issue. The result reveals that the accuracy scores of the models have fallen. However, the drop in accuracy is irrelevant in this case, as the dataset contains 68% fallers and 32% non-fallers. When dividing them into training and testing sets, the majority of the train and predicted classes will be fallers, since the non-fallers have only a few data points. As a result, accuracy will naturally be high because the most trained and predicted set is a faller.

**Table 4:** Accuracy comparison.

	AdaBoost	Bagging	CatBoost	Decision Tree	KNN	**LightGBM**	MLP	NB	RF	SVM	Voting	XGBoost
Experiment 1	0.81	0.89	0.89	0.81	0.96	**0.96**	0.96	0.81	0.81	0.89	0.85	0.81
Experiment 1 without SMOTE	0.95	1.00	1.00	0.95	1.00	**1.00**	1.00	0.95	0.95	1.00	1.00	0.90
Experiment 1 without enhancement	0.95	1.00	1.00	0.95	0.80	**1.00**	1.00	0.95	0.95	0.70	1.00	0.90
Experiment 1 without feature scaling	0.81	0.89	0.89	0.81	0.78	**0.96**	0.93	0.81	0.81	0.78	0.85	0.81
Experiment 2	0.81	0.89	0.89	0.89	0.93	**0.96**	0.96	0.81	0.81	0.89	0.89	0.81
Experiment 2 without SMOTE	0.90	1.00	0.95	0.95	1.00	**1.00**	1.00	0.95	0.90	1.00	0.90	0.95
Experiment 2 without enhancement	0.90	1.00	0.95	0.95	0.70	**1.00**	1.00	0.95	0.90	0.70	0.90	0.95
Experiment 2 without feature scaling	0.81	0.89	0.89	0.89	0.78	**0.96**	0.85	0.81	0.81	0.74	0.89	0.81

The bold values represent the accuracy score achieved by our proposed LightGBM model, as well as the highest score obtained in every experiment.

**Figure 11:**
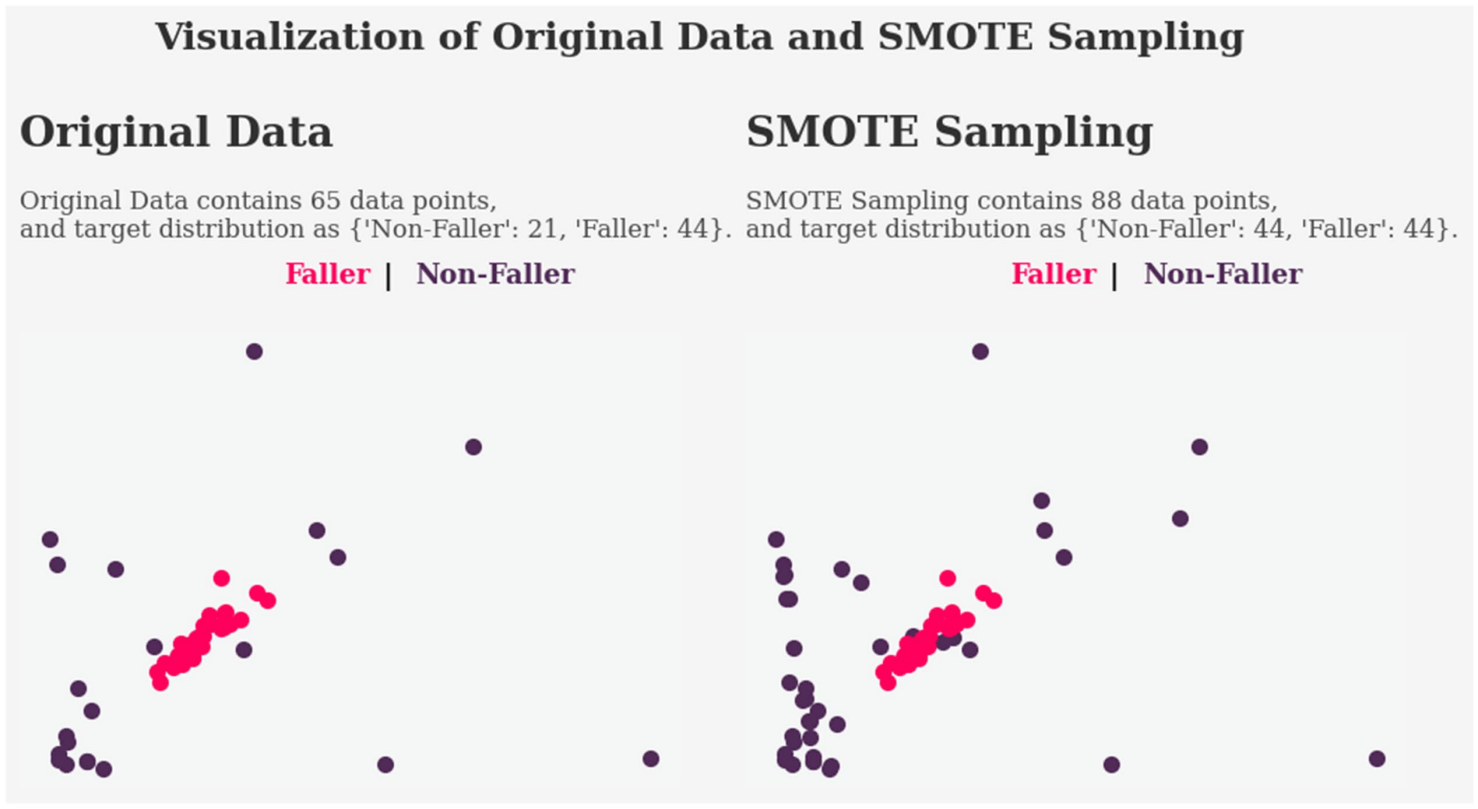
Comparison of original data and SMOTE sampled data.

Figure 11 represents the before and after applying SMOTE sampling method, where the feature space of the non-faller data has increased significantly. SMOTE has allowed the model to be trained and evaluated evenly, while also having balanced and larger data that can help in achieving a better generalization of the model.

### 4.6 Assessing the impact of feature scaling

**Figure 12:**
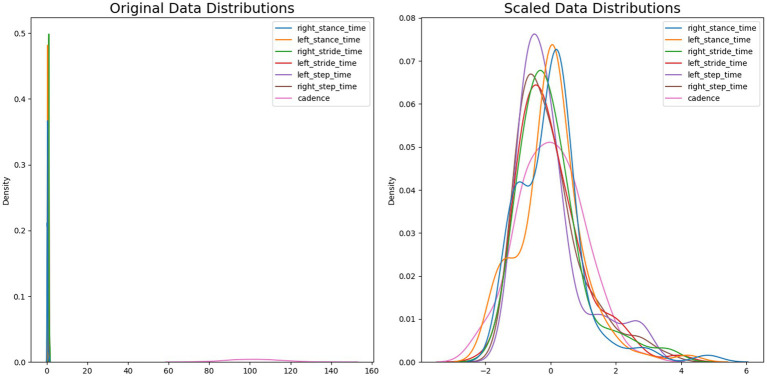
Comparison of original data distributions and scaled data distributions.

Figure 12 illustrates the effect of applying a standard scaler to the dataset. The x-axis displays the feature value, while the y-axis displays the density of the feature value. Each feature represents a different color bar. The original data distribution contains a wide range of feature values, ranging from 0 to 160, as well as a density value of around 0 to 0.5. After applying the standard scaler, the ranging value from both decreases noticeably, with the feature value scaled between −2 and 6 and a density value of 0 to 0.08.

Table 4 demonstrates a significant impact of accuracies before and after feature scaling. However, some algorithms exhibit sensitivity to feature scale due to their distance-based nature, resulting in an increase or decrease in performance. Nonetheless, we intend to use feature scaling to prevent any bias in our algorithm toward a particular feature.

### 4.7 Classification and performance evaluation

In the classification phase, the entire size of the dataset was 88, including 65 of the actual data and 23 oversampled data from the minority class (non-fallers). The dataset was then shuffled, divided into training and testing sets with a 70:30 ratio, and stratified to ensure equal proportions in each class, resulting in 61 instances for training and 27 instances for testing (13 non-faller, 14 fallers). Twelve classifiers, namely SVM, DT, RF, LightGBM, XGBoost, CatBoost, AdaBoost, KNN, Voting, NB, MLP, and Bagging were used. To obtain the best set of model parameters, hyperparameter tuning was performed using the sklearn library’s ParameterGrid.

#### 4.7.1 Experiment 1

**Figure 13:**
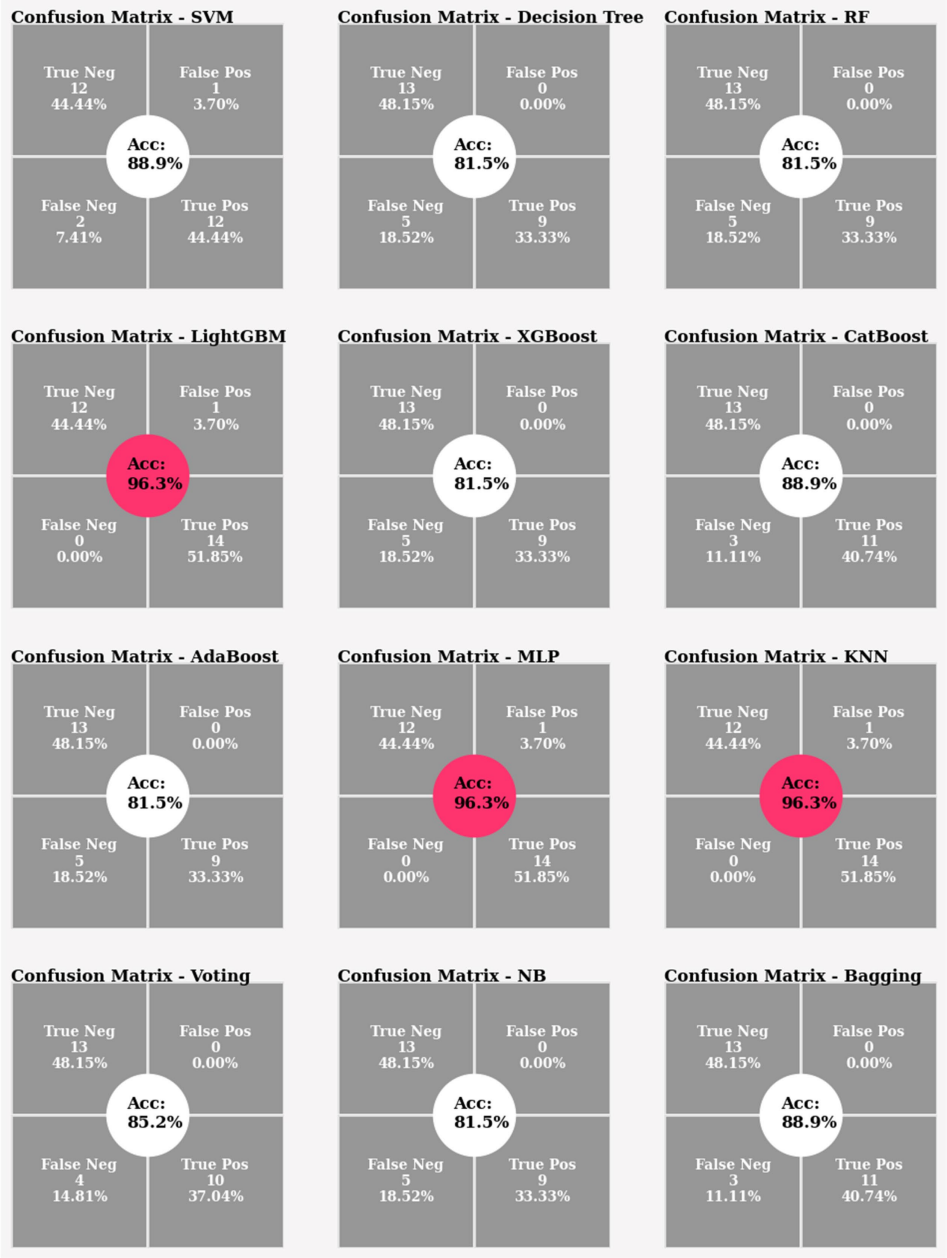
Confusion matrix and accuracy comparison for Experiment 1.

In the first experiment, classifiers were achieved in the range between 81% to 96% accuracy. However, the remarkable 96% accuracy was attained by the LightGBM, MLP and KNN classifiers (Figure 13).

#### 4.7.2 Experiment 2

**Figure 14:**
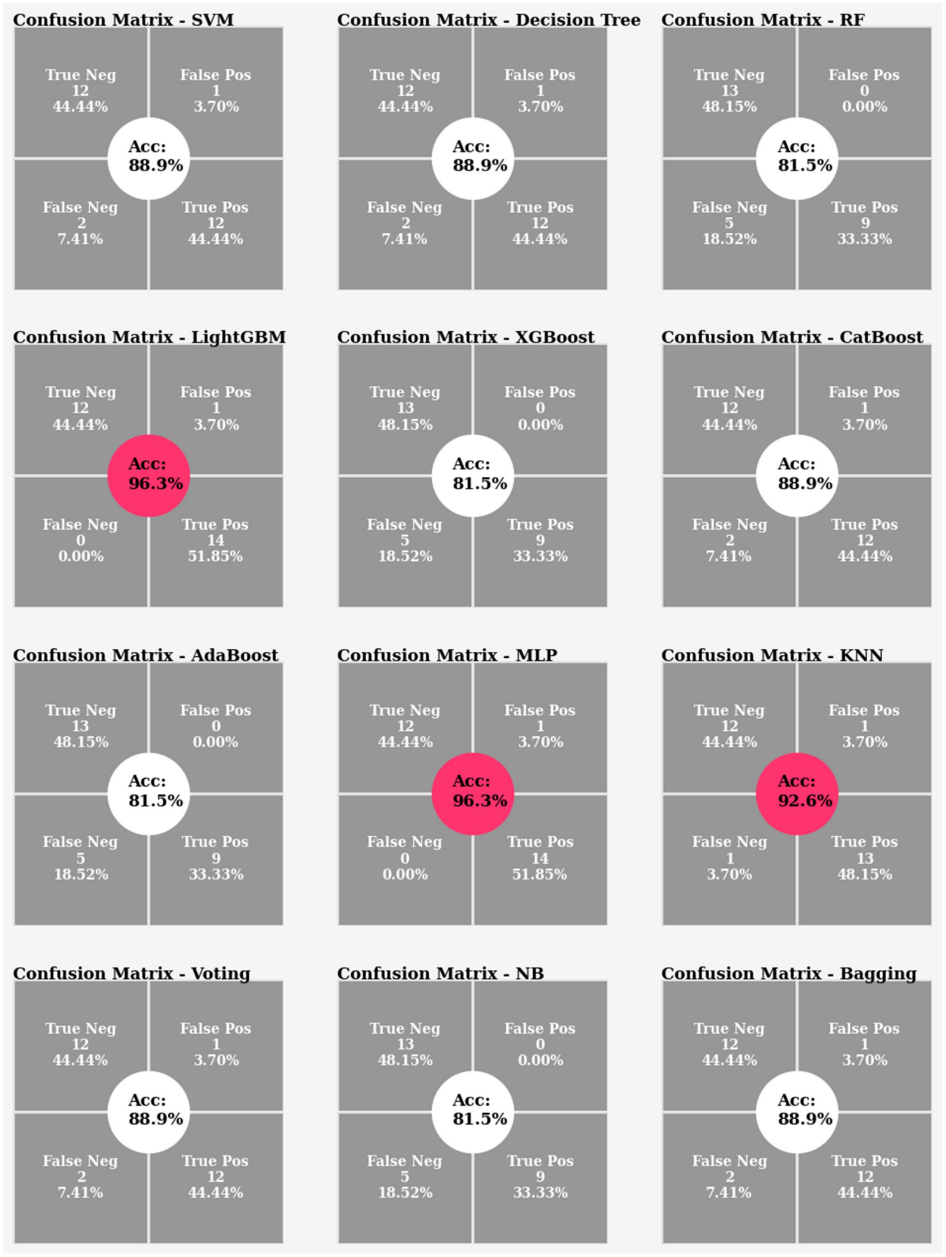
Confusion matrix and accuracy comparison for Experiment 2.

**Figure 15:**
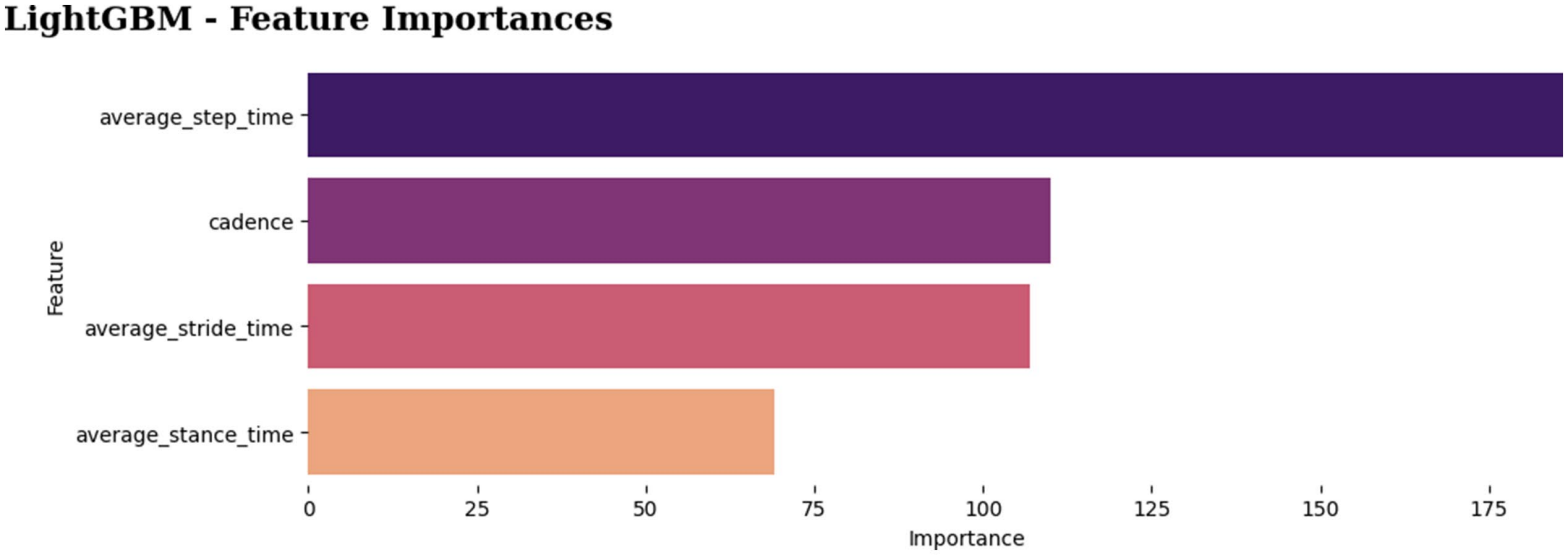
Feature importances in LightGBM model.

In Experiment 2, which used the average gait features, the classifiers were able to reach 81% to 96% accuracy. However, the remarkable 96% accuracy was attained by the MLP and LightGBM classifiers (Figure 14). Figure 15 depicts the feature importance analysis, with step time and cadence receiving high importance scores, indicating a significant impact on the model’s predictions.

In summary, the machine learning models achieved good performances of 81 to 96% accuracy, demonstrating a promising performance of the machine learning models in the fall’s prediction. In terms of performance and computational time, the proposed classifier, LightGBM, surpassed the others by having the fastest computing time and the greatest accuracy score in classification task. Furthermore, the two most important factors, step time and cadence, appear to be closely related to the risk of falling.

## 5 Discussion

This section summarizes some interesting findings discovered in the study:

- Effectiveness of machine learning approaches: Machine learning algorithms demonstrate high performance in classification tasks, with accuracies of 81% or higher. Notably, the LightGBM classifier shows superior results with 96% accuracy in both experiments and takes less computational time.
- Risk Factor Identification: The study reveals that longer cadence, as well as shorter step times, may raise the risk of falls in individuals.
- Distinctive Gait Features of Faller and Non-Faller: This study has shown that gait characteristics are useful for predicting falls. According to the statistics, fallers appear to have a longer stance time, stride time, and cadence, but a shorter step time. However, we cannot just decide if people with longer stance times will fall. Similar to a puzzle, every feature has a connection with others which helps to produce the final output.

## 6 Conclusion

In conclusion, this study has made significant strides in the realm of fall risk prediction by harnessing the power of artificial intelligence and gait analysis. The dual-experimental approach, analyzing both individual and averaged gait features, has underscored the versatility and robustness of AI methodologies in capturing the nuances of human gait dynamics. The suggested machine learning model, LightGBM demonstrates remarkable efficacy and performance in classification, attaining 96% accuracy in both experiments. Moreover, several significant discoveries were made during the classification phase, such as the possibility that longer cadence as well as shorter step times could increase an individual’s risk of falling. Despite the model showing promising performance in prediction tasks, its potential to be applied to real-world scenarios is limited due to overfitting issue caused by the limited dataset, reliance on synthetic data, and lack of diversity in the dataset. Therefore, future research should focus on expanding the diversity of datasets, exploring deeper machine learning and deep learning models, and investigating the real-world applicability of these predictive tools in clinical settings. Another aspiring approach for future research is to implement SHAP values into the experiment to gain an in-depth understanding of how these features contribute to the model’s prediction.

## Data Availability

The datasets presented in this study can be found in online repositories. The names of the repository/repositories and accession number(s) can be found at: https://www.kaggle.com/datasets/limzhekhae1008/mmu-fall-risk-prediction-mmu-frip-dataset.
